# Some inequalities for the Fan product of *M*-tensors

**DOI:** 10.1186/s13660-018-1853-1

**Published:** 2018-09-24

**Authors:** Gang Wang, Yanan Wang, Yuan Zhang

**Affiliations:** 1 School of Management Science, Qufu Normal University, Rizhao, P.R. China; 2 School of Management and Economics, Beijing Institute of Technology, Beijing, P.R. China

**Keywords:** 15A18, 15A69, *M*-tensors, Fan product of *M*-tensors, Minimum eigenvalue

## Abstract

In this paper, we investigate some inequalities for the Fan product of *M*-tensors. We propose exact characterizations of *M*-tensors and establish some inequalities on the minimum eigenvalue for the Fan product of two *M*-tensors. Furthermore, the inclusion relations among them are discussed. Numerical examples show the validity of the conclusions.

## Introduction

Let $\mathbb{C} (\mathbb{R})$ be the set of all complex (real) numbers, $\mathbb{R}_{+} (\mathbb{R}_{++})$ be the set of all nonnegative (positive) numbers, $\mathbb{C}^{n} (\mathbb{R}^{n})$ be the set of all dimension *n* complex (real) vectors, and $\mathbb{R}_{+}^{n} (\mathbb{R}_{++}^{n})$ be the set of all dimension *n* nonnegative (positive) vectors. An *m*th order *n*-dimensional tensor $\mathcal{A}=(a_{i_{1}i_{2}\dots i_{m}})$ is a higher-order generalization of matrices, which consists of $n^{m}$ entries:

$$a_{i_{1}i_{2}\dots i_{m}}\in\mathbb{R}, \quad i_{k}\in N= \{1,2,\ldots,n\}, k=1,2,\ldots,m. $$

$\mathcal{A}$ is called nonnegative (positive) if $a_{{i_{1}}{i_{2}}\ldots {i_{m}}}\in\mathbb{R}_{+}$ ($a_{{i_{1}}{i_{2}}\ldots{i_{m}}}\in\mathbb{R}_{++}$).

Tensors have many similarities with matrices and many related results of matrices such as determinant, eigenvalue, and algorithm theory can be extended to higher order tensors [1–3]. Furthermore, structured matrices such as nonnegative matrices, *H*-matrices and *M*-matrices can also be extended to higher order tensors and these are becoming the focus of recent tensor research [4–26]. In particular, *M*-tensors play important roles in the stability study of nonlinear autonomous systems via Lyapunov’s direct method in automatic control [27–29] and spectral hypergraph theory [3, 30, 31].

On the other hand, Fan product of *M*-matrices and Hadamard product of nonnegative matrices are significant for practical problems, such as the weak minimum principle in partial differential equations, products of integral equation kernels, characteristic functions in probability theory, the study of association schemes in combinatorial theory, and so on (see [32]). Some inequalities on the spectral radius for the Hadamard product of two nonnegative matrices and some inequalities on the minimum eigenvalue for the Fan product of two *M*-matrices can be found in [33–37]. Recently, Sun et al. [14] investigated some inequalities for the Hadamard product of tensors and obtained some bounds on the spectral radius, and used them to estimate the spectral radius of a directly weighted hypergraph. It is well known that an *M*-tensor is defined based on a *Z*-tensor and its algebra properties can be explored using the spectral theory of nonnegative tensors [23]. Motivated by these observations, we expect to establish sharp lower bounds on the minimal eigenvalue for the Fan product of two *M*-tensors and discuss some inclusion relations among them.

The remaining of this paper is organized as follows. In Sect. 2, we introduce important notation and recall some preliminary results on tensor analysis. In Sect. 3, based on exact characterizations of *M*-tensors, we give a lower bound on the minimum eigenvalue for the Fan product of two *M*-tensors. An improved result is established for irreducible nonnegative tensors by the ratio of the smallest and largest values of a Perron vector. Finally, making use of the information of the absolute maximum in the off-diagonal elements, we obtain a new lower bound on the minimum eigenvalue for the Fan product. With numerical examples, we exhibit the efficiency of the results given in Theorems 1–3.

## Notation and preliminaries

We start this section with some fundamental notions and properties developed in tensor analysis [1, 3], which are needed in the subsequent analysis.

### Definition 1

Let $\mathcal{A}$ be an *m*-order *n*-dimensional tensor. Assume that $\mathcal{A}x^{m-1}$ is not identical to 0. We say that $(\lambda,x)\in\mathbb{C}\times(\mathbb{C}^{n}\setminus \{0\})$ is an eigenvalue–eigenvector of $\mathcal{A}$ if

$$\mathcal{A}x^{m-1}=\lambda x^{[m-1]}, $$

where $(\mathcal{A} x^{m-1})_{i}=\sum^{n}_{i_{2},\ldots, i_{m}=1}a_{{i}{i_{2}}\dots{i_{m}}}x_{i_{2}}\cdots x_{i_{m}}$, $x^{[m-1]}=[x^{m-1}_{1},x^{m-1}_{2},\ldots,x^{m-1}_{n}]^{T}$, and $(\lambda,x)$ is called an *H*-eigenpair if they are both real.

### Definition 2

Let $\mathcal{A}$ and $\mathcal{I}$ be *m*-order *n*-dimensional tensors.

(i) We call $\sigma(\mathcal{A})$ as the set of all eigenvalues of $\mathcal{A}$. Assume $\sigma(\mathcal{A})\neq\emptyset$. Then the spectral radius of $\mathcal{A}$ is denoted by
  $$\rho(\mathcal{A})=\max\bigl\{ |\lambda|: \lambda\in\sigma(\mathcal{A})\bigr\} . $$
  Meanwhile, we use $\tau(\mathcal{A})$ to denote the minimal value of the real part of eigenvalues of $\mathcal{A}$.
(ii) We call a tensor $\mathcal{A}$ reducible if there exists a nonempty proper index subset $I\subset\{1,2,\dots,n\}$ such that
  $$a_{{i_{1}}{i_{2}}\dots{i_{m}}}=0, \quad \forall i_{1}\in I, i_{2}, \dots,i_{m}\notin I. $$
  If $\mathcal{A}$ is not reducible, then we call $\mathcal{A}$ irreducible.
(iii) We call a nonnegative matrix $GM(\mathcal{A})$ the representation associated to a nonnegative tensor $\mathcal{A}$, if the $(i, j)$th entry of $GM(\mathcal{A})$ is defined to be the sum of $a_{ii_{2} i_{3}\ldots i_{m}}$ with indices $j\in\{i_{2}, i_{3}, \ldots, i_{m}\} $. We call a tensor $\mathcal{A}$ weakly reducible, if its representation $GM(\mathcal{A})$ is reducible. It is weakly irreducible if it is not weakly reducible.
(iv) We call $\mathcal{I}$ is a unit tensor whose entries are
  $$\delta_{{i_{1}}{i_{2}}\ldots{i_{m}}}= \textstyle\begin{cases} 1, & \text{if } i_{1}=i_{2}= \cdots=i_{m}, \\ 0, & \text{otherwise}. \end{cases} $$

It is noted that the spectral radius $\rho(\mathcal{A})$ is the largest *H*-eigenvalue for the nonnegative tensor [4] and $\tau (\mathcal{A})$ is smallest *H*-eigenvalue for the *M*-tensor [23].

The Perron–Frobenius theorem for nonnegative weakly irreducible tensors has been established in [9, 11, 22].

### Lemma 1

*Let* $\mathcal{A}$ *be a weakly irreducible nonnegative tensor of order* *m* *and dimension* *n*. *Then the following results hold*:

(i) $\mathcal{A}$ *has a positive eigenpair* $(\lambda,x)$ *and* *x* *is unique up to a multiplicative constant*.
(ii) $$\min_{x\in\mathbb{R}^{n}_{++}}\max_{1\leq i\leq n}\frac{(\mathcal {A}x^{m-1})_{i}}{{{x_{i}}}^{[m-1]}}=\rho( \mathcal{A})=\max_{x\in\mathbb {R}^{n}_{+}\setminus \{0\}}\min_{x_{i}\neq0, 1\leq i\leq n} \frac{(\mathcal {A}x^{m-1})_{i}}{{{x_{i}}}^{[m-1]}}. $$

The following specially structured tensors are extended from matrices [8, 23].

### Definition 3

Let $\mathcal{A}$ and $\mathcal{U}$ be *m*-order *n*-dimensional tensors.

(i) We call $\mathcal{A}$ is a *Z*-tensor if all its off-diagonal entries are nonpositive.
(ii) We call $\mathcal{A}$ is an *M*-tensor if there exist a nonnegative tensor $\mathcal{U}$ and a positive real number $\eta\geq \rho(\mathcal{U})$ such that
  $$\mathcal{A}=\eta\mathcal{I}-\mathcal{U}. $$
  If $\eta> \rho(\mathcal{U})$, then $\mathcal{A}$ is called a strong *M*-tensor.
(iii) We call $\mathcal{A}$ is a weakly irreducible *M*-tensor if $\mathcal{U}$ is weakly irreducible.
(iv) Assume $\mathcal{A}$ and $\mathcal{B}$ are *M*-tensors. The Fan product of $\mathcal{A}$ and $\mathcal{B}$ is denoted by $\mathcal {A}\star\mathcal{B}=\mathcal{D}=(d_{i_{1} i_{2}\ldots i_{m}})$ and defined by
  $${d_{{i_{1}}{i_{2}} \ldots{i_{m}}}} = { \textstyle\begin{cases} {{a_{i \ldots i}}{b_{i \ldots i}}}, & {{i_{1}} = {i_{2}} = \cdots={i_{m}} = i}, \\ { - |{a_{{i_{1}}{i_{2}} \ldots{i_{m}}}}{b_{{i_{1}}{i_{2}} \ldots{i_{m}}}}|}, & \text{otherwise}. \end{cases}\displaystyle } $$

It is easy to see that all the diagonal entries of an *M*-tensor are nonnegative [23], and the (strong) *M*-tensor is closely linked with the diagonal dominance defined below.

### Definition 4

An *m*-order *n*-dimensional tensor $\mathcal{A}$ is called diagonally dominant if

$$|a_{i\ldots i}|\geq\sum_{\delta_{{i}{i_{2}}\ldots {i_{m}}}=0}|a_{i{i_{2}}\ldots{i_{m}}}|, \quad \forall i\in N; $$

$\mathcal{A}$ is called strictly diagonally dominant if the strict inequalities hold for all $i\in N$.

Define a positive diagonal matrix $D =\operatorname{diag}(d_{1},\ldots, d_{n})$ and set

1 $$ \mathcal{B}=(b_{i_{1} i_{2} \ldots i_{m}})=\mathcal{A}\cdot D^{-(m-1)} \overbrace{D\cdots D}^{m-1}=\bigl(a_{i_{1}\dots i_{m}}d^{-(m-1)}_{i_{1}}d_{i_{2}} \cdots d_{i_{m}}\bigr). $$

We obtain the following necessary and sufficient condition for identifying *M*-tensors.

### Lemma 2

([23])

*Suppose* $\mathcal{A}$ *is a weakly irreducible* *Z*-*tensor and its all diagonal elements are nonnegative*. *Then* $\mathcal{A}$ *is an* (*strong*) *M*-*tensor if and only if there exists a positive diagonal matrix* *D* *such that* $\mathcal{B}$ *defined in* (1) *is* (*strictly*) *diagonally dominant*.

## Some inequalities on the minimum eigenvalue for the Fan product

In this section, we shall give lower bounds on the minimum eigenvalue for the Fan product. Firstly, we establish characterizations of *M*-tensors.

### Lemma 3

*Let* $\mathcal{Q}$ *be a weakly irreducible* *M*-*tensor of order* *m* *and dimension* *n*. *If* $\mathcal{Q}z^{m-1}\geq kz^{[m-1]}$ *for a vector* $z\in R^{n}_{++}$ *and a real number* *k*, *then* $k\leq\tau(\mathcal{Q})$.

### Proof

Since $\mathcal{Q}$ is an *M*-tensor, there exists a nonnegative tensor $\mathcal{U}$ such that

2 $$ \mathcal{Q}=\lambda\mathcal{I}-\mathcal{U}, $$

where *λ* is a nonnegative real number and $\lambda\geq\rho (\mathcal{U})$. It is easy to see that $\tau(\mathcal{Q})=\lambda-\rho (\mathcal{U})$. Furthermore, $\rho(\mathcal{U})=\lambda-\tau(\mathcal{Q})$. Taking into account that $\mathcal{Q}$ is weakly irreducible, we deduce that $\mathcal{U}$ is weakly irreducible. From the assumption and (2), we have

$$(\lambda\mathcal{I}-\mathcal{U})z^{m-1}\geq k z^{[m-1]}, $$

that is,

$$(\lambda-k)z^{[m-1]}\geq\mathcal{U} z^{m-1}. $$

It follows from Lemma 1 that

$$\lambda-k\geq\rho(\mathcal{U})= \lambda-\tau(\mathcal{Q}). $$

So, $\tau(\mathcal{Q})\geq k$. □

### Lemma 4

*Let* $\mathcal{P}$, $\mathcal{Q}$ *be two* *M*-*tensors of order* *m* *and dimension* *n*. *Then* $\mathcal{P}\star\mathcal{Q}$ *is an* *M*-*tensor*. *Furthermore*, *if* $\mathcal{P}$, $\mathcal{Q}$ *are strong* *M*-*tensors*, *then* $\mathcal{P}\star\mathcal{Q}$ *is a strong* *M*-*tensor*.

### Proof

By the definition of $\mathcal{P}\star\mathcal{Q}$, it holds that

$$\mathcal{P}\star\mathcal{Q}= \textstyle\begin{cases} p_{i\ldots i}q_{i\ldots i},&\mbox{if } i_{2}=i_{3}=\cdots=i_{m}=i, \\ -|p_{i i_{2}\ldots i_{m}}q_{i i_{2}\ldots i_{m}}|,&\mbox{otherwise}. \end{cases} $$

Since $\mathcal{P}$, $\mathcal{Q}$ are *M*-tensors, by Lemma 1, there exist positive diagonal matrices *C*, *D* such that

$$\mathcal{A}=\mathcal{P}\cdot C^{-(m-1)}\overbrace{C\cdots C}^{m-1},\qquad \mathcal{B}=\mathcal{Q}\cdot D^{-(m-1)}\overbrace{D \cdots D}^{m-1} $$

with

$$a_{i_{1}\dots i_{m}}=p_{i_{1}\dots i_{m}}c^{-(m-1)}_{i_{1}}c_{i_{2}} \cdots c_{i_{m}},\qquad b_{i_{1}\dots i_{m}}=q_{i_{1}\dots i_{m}}d^{-(m-1)}_{i_{1}}d_{i_{2}} \cdots d_{i_{m}}. $$

Specifically,

$$a_{i\ldots i}=p_{i\ldots i},\qquad b_{i\ldots i}=q_{i\ldots i}. $$

Taking into account that $\mathcal{A}$ and $\mathcal{B}$ are diagonally dominant, we conclude that

$$\begin{aligned}& {|p_{i\ldots i}|=|a_{i\ldots i}|\geq\sum_{\delta_{{i}{i_{2}}\ldots {i_{m}}}=0}|p_{i i_{2}\dots i_{m}}|c^{-(m-1)}_{i}c_{i_{2}} \cdots c_{i_{m}}}, \\& {|q_{i\ldots i}|=|b_{i\ldots i}|\geq\sum_{\delta_{{i}{i_{2}}\ldots {i_{m}}}=0}|q_{ii_{2}\dots i_{m}}|d^{-(m-1)}_{i}d_{i_{2}} \cdots d_{i_{m}}}. \end{aligned}$$

Furthermore, it holds that

3 $$\begin{aligned} |p_{i\ldots i}q_{i\ldots i}| =&|a_{i\ldots i}b_{i\ldots i}| \\ \geq&\sum_{\delta_{{i}{i_{2}}\ldots{i_{m}}}=0}\bigl(|p_{i i_{2}\dots i_{m}}|c^{-(m-1)}_{i}c_{i_{2}} \dots c_{i_{m}}\bigr)\sum_{\delta_{{i}{i_{2}}\ldots {i_{m}}}=0} \bigl(|q_{i i_{2}\dots i_{m}}|d^{(-m-1)}_{i}d_{i_{2}}\cdots d_{i_{m}}\bigr) \\ \geq&\sum_{\delta_{{i}{i_{2}}\ldots{i_{m}}}=0}|p_{i i_{2}\dots i_{m}}|c^{-(m-1)}_{i}c_{i_{2}} \cdots c_{i_{m}}|q_{i i_{2}\dots i_{m}}|d^{-(m-1)}_{i}d_{i_{2}} \cdots d_{i_{m}} \\ =&\sum_{\delta_{{i}{i_{2}}\ldots{i_{m}}}=0}|p_{i i_{2}\dots i_{m}}q_{i i_{2}\dots i_{m}}| (c_{i} d_{i})^{-(m-1)}c_{i_{2}}d_{i_{2}} \cdots c_{i_{m}}d_{i_{m}}. \end{aligned}$$

Hence, it follows from (3) that there exists a positive diagonal matrix $U=\operatorname{diag} (c_{1} d_{1},c_{2} d_{2}, \ldots,c_{n} d_{n})$ such that

$$|p_{i\ldots i}q_{i\ldots i}|\geq\sum_{\delta _{{i}{i_{2}}\ldots{i_{m}}}=0}p_{i i_{2}\dots i_{m}}q_{i i_{2}\dots i_{m}} {u_{i}}^{-(m-1)}u_{i_{2}}\cdots u_{i_{m}}. $$

It follows from Lemma 2 that $\mathcal{P}\star\mathcal{Q}$ is an *M*-tensor. By a similar argument as for the first conclusion, we can obtain the second conclusion. □

Suppose that $\mathcal{P}=(p_{i_{1} i_{2} \ldots i_{m}})$ is a strong *M*-tensor of order *m* and dimension *n*. Set $\mathcal{N}=\mathcal {D}-\mathcal{P}$, where $\mathcal{D}$ denotes the diagonal tensor of the same order, dimension and diagonal entries as $\mathcal{P}$. Note that $p_{ii\ldots i}>0$ for $i\in N$ when $\mathcal{P}$ is a strong *M*-tensor. Define $J_{\mathcal{P}}=\mathcal{D}^{-1}\mathcal{N}$. Obviously, $J_{\mathcal{P}}$ is nonnegative. The following result characterizes $J_{\mathcal{P}}$ in terms of the spectral radius.

### Lemma 5

*Suppose that* $\mathcal{P}=(p_{i_{1} i_{2} \ldots i_{m}})$ *is a strong* *M*-*tensor of order* *m* *and dimension* *n*. *Then*

$$\rho(J_{\mathcal{P}})\geq1-\frac{\tau(\mathcal{P})}{\min_{1\leq i\leq n}p_{i i \ldots i}}. $$

*Furthermore*, *if* $\mathcal{P}$ *is weakly irreducible*, *then*

$$\rho(J_{\mathcal{P}})\leq1-\frac{\tau(\mathcal{P})}{\max_{1\leq i\leq n}p_{i i \ldots i}}. $$

### Proof

Let $\mathcal{P}=(p_{i_{1} i_{2} \ldots i_{m}})$ be a strong *M*-tensor. Then there exists a positive vector $u= (u_{i})$ such that

$$p_{i\ldots i}u^{[m-1]}_{i}+\sum _{\delta_{{i}{i_{2}}\ldots {i_{m}}}=0}p_{i{i_{2}}\ldots{i_{m}}}u_{i_{2}}\cdots u_{i_{m}}= \tau(\mathcal {P})u^{[m-1]}_{i}, $$

that is,

4 $$ \frac{\sum_{\delta_{{i}{i_{2}}\ldots{i_{m}}}=0}p_{i{i_{2}}\ldots {i_{m}}}u_{i_{2}}\cdots u_{i_{m}}}{p_{i\ldots i}u^{[m-1]}_{i}}=\frac{\tau (\mathcal{P})}{p_{i\ldots i}} -1. $$

Since the tensor $J_{\mathcal{P}}$ is nonnegative, by Lemma 1 and (4), we have

5 $$\begin{aligned} \rho(J_{\mathcal{P}})&= \max_{x\in\mathbb{R}^{n}_{+}\setminus \{0\}}\min_{x_{i}\neq0, 1\leq i\leq n} \frac{(J_{\mathcal {P}}x^{m-1})_{i}}{{{x_{i}}}^{[m-1]}}\geq\min_{1\leq i\leq n}\frac {(J_{\mathcal{P}}u^{m-1})_{i}}{{{u_{i}}}^{[m-1]}} \\ & = \min_{1\leq i\leq n} \frac{\sum_{\delta_{{i}{i_{2}}\ldots {i_{m}}}=0}-p_{i{i_{2}}\ldots{i_{m}}}u_{i_{2}}\ldots u_{i_{m}}}{p_{i\ldots i}u^{[m-1]}_{i}}= \min_{1\leq i\leq n} \biggl(1-\frac{\tau(\mathcal {P})}{p_{i\ldots i}}\biggr) \\ &=1-\frac{\tau(\mathcal{P})}{\min_{1\leq i\leq n}p_{i i \ldots i}}. \end{aligned}$$

Furthermore, $J_{\mathcal{P}}$ is weakly irreducible when $\mathcal{P}$ is weakly irreducible. From Lemma 1 and (4), it holds that

6 $$\begin{aligned} \rho(J_{\mathcal{P}})& = \min_{x\in\mathbb{R}^{n}_{++}}\max_{1\leq i\leq n} \frac{(J_{\mathcal{P}}x^{m-1})_{i}}{{{x_{i}}}^{[m-1]}}\leq\max_{1\leq i\leq n}\frac{(J_{\mathcal{P}}u^{m-1})_{i}}{{{u_{i}}}^{[m-1]}} \\ & = \max_{1\leq i\leq n} \frac{\sum_{\delta_{{i}{i_{2}}\ldots {i_{m}}}=0}-p_{i{i_{2}}\ldots{i_{m}}}u_{i_{2}}\cdots u_{i_{m}}}{p_{i\ldots i}u^{[m-1]}_{i}}=\max_{1\leq i\leq n} \biggl(1-\frac{\tau(\mathcal {P})}{p_{i\ldots i}}\biggr) \\ &=1-\frac{\tau(\mathcal{P})}{\max_{1\leq i\leq n}p_{i i \ldots i}}. \end{aligned}$$

 □

The following example shows that the bound of Lemma 5 is tight.

### Example 1

Let $\mathcal{P}=(p_{ijk})$ be a tensor of order 3 and dimension 3 with elements defined as follows:

$$p_{ijk}= \textstyle\begin{cases} p_{111}=p_{222}=p_{333}=3, \\ p_{ijk}=-\frac{1}{4}, \quad \text{otherwise}. \end{cases} $$

By computations, we get $\tau(\mathcal{P})=1$ and

$$\rho(J_{\mathcal{P}})=1-\frac{\tau(\mathcal{P})}{\min_{1\leq i\leq n}p_{i i \ldots i}}= 1-\frac{\tau(\mathcal{P})}{\max_{1\leq i\leq n}p_{i i \ldots i}}= \frac{2}{3}. $$

Based on the characterizations of *M*-tensors, we can immediately obtain these bounds from the following result.

### Theorem 1

*If* $\mathcal{P}$ *and* $\mathcal{Q}$ *are two strong* *M*-*tensors of order* *m* *and dimension* *n*, *then*

7 $$ \tau(\mathcal{P}\star\mathcal{Q})\geq\bigl(1-\rho(J_{\mathcal{P}}) \rho (J_{\mathcal{Q}})\bigr)\min_{1\leq i\leq n}(p_{i\ldots i}q_{i\ldots i}). $$

### Proof

Let us distinguish two cases.

Case 1. $\mathcal{P}$ and $\mathcal{Q}$ are both weakly irreducible. It follows from Lemma 4 that $\mathcal{P}\star\mathcal{Q}$ is a strong *M*-tensor. Since $J_{\mathcal{P}}$ and $J_{\mathcal{Q}}$ are weakly irreducible nonnegative tensors, from Lemma 1, there exist two positive vectors *u*, *v* such that

$$\rho(J_{\mathcal{P}})u^{[m-1]}_{i}=J_{\mathcal{P}}u^{m-1}, \qquad \rho (J_{\mathcal{Q}})v^{[m-1]}_{i}=J_{\mathcal{Q}}v^{m-1}, $$

equivalently,

8 $$ \frac{\sum_{\delta_{{i}{i_{2}}\ldots{i_{m}}}=0}|p_{i{i_{2}}\ldots {i_{m}}}|u_{i_{2}}\cdots u_{i_{m}}}{p_{i\ldots i}u^{[m-1]}_{i}}=\rho(J_{\mathcal {P}}),\qquad \frac{\sum_{\delta_{{i}{i_{2}}\ldots{i_{m}}}=0}|q_{i{i_{2}}\ldots {i_{m}}}|v_{i_{2}}\cdots v_{i_{m}}}{q_{i\ldots i}v^{[m-1]}_{i}}= \rho(J_{\mathcal{Q}}). $$

Let $z=(z_{i})$, where $z_{i}= u_{i} v_{i} \in\mathbb{R}_{++}$ for $i\in N$. Setting $\mathcal{U}=\mathcal{P} \star\mathcal{Q}$, for $i \in N$, we obtain

9 $$\begin{aligned} &\bigl(\mathcal{U}z^{m-1}\bigr)_{i} \\ &\quad = p_{i\ldots i}q_{i\ldots i}u^{[m-1]}_{i} v^{[m-1]}_{i} -\sum_{\delta_{{i}{i_{2}}\ldots{i_{m}}}=0} \vert p_{i{i_{2}}\ldots {i_{m}}} \vert u_{i_{2}}\cdots u_{i_{m}} \vert q_{i{i_{2}}\ldots{i_{m}}} \vert v_{i_{2}}\cdots v_{i_{m}} \\ &\quad \geq p_{i\ldots i}q_{i\ldots i}u^{[m-1]}_{i} v^{[m-1]}_{i}- \sum_{\delta _{{i}{i_{2}}\ldots{i_{m}}}=0}\bigl( \vert p_{i{i_{2}}\ldots{i_{m}}} \vert u_{i_{2}}\cdots u_{i_{m}}\bigr) \sum_{\delta_{{i}{i_{2}}\ldots{i_{m}}}=0}\bigl( \vert q_{i{i_{2}}\ldots {i_{m}}} \vert v_{i_{2}}\cdots v_{i_{m}}\bigr) \\ &\quad = p_{i\ldots i}q_{i\ldots i}u^{[m-1]}_{i} v^{[m-1]}_{i} \\ &\qquad {}\times\biggl(1-\frac{\sum_{\delta_{{i}{i_{2}}\ldots{i_{m}}}=0} \vert p_{i{i_{2}}\ldots{i_{m}}} \vert u_{i_{2}}\cdots u_{i_{m}}}{p_{i\ldots i}u^{[m-1]}_{i}}\frac{\sum_{\delta_{{i}{i_{2}}\ldots {i_{m}}}=0} \vert q_{i{i_{2}}\ldots{i_{m}}} \vert v_{i_{2}}\cdots v_{i_{m}}}{q_{i\ldots i}v^{[m-1]}_{i}} \biggr) \\ &\quad =p_{i\ldots i}q_{i\ldots i}u^{[m-1]}_{i} v^{[m-1]}_{i} \bigl(1-\rho(J_{\mathcal {P}}) \rho(J_{\mathcal{Q}})\bigr)=p_{i\ldots i}q_{i\ldots i} \bigl(1-\rho (J_{\mathcal{P}}) \rho(J_{\mathcal{Q}})\bigr)z^{[m-1]}_{i}. \end{aligned}$$

It follows from Lemma 3 and (9) that

$$\tau(\mathcal{P}\star\mathcal{Q})\geq\bigl(1-\rho(J_{\mathcal{P}}) \rho (J_{\mathcal{Q}})\bigr)\min_{1\leq i\leq n}(p_{i\ldots i}q_{i\ldots i}). $$

Case 2. Either $\mathcal{P}$ or $\mathcal{Q}$ is weakly reducible. Let $\mathcal{S}$ be a tensor of order *m* and dimension *n* with

$$s_{i i_{2}\ldots i_{m}}= \textstyle\begin{cases} 1, & \text{if } i_{2}=i_{3}=\cdots=i_{m} \neq i, \\ 0, & \text{otherwise}. \end{cases} $$

Then both $\mathcal{P}- \epsilon\mathcal{S}$ and $\mathcal{Q}- \epsilon\mathcal{S}$ are weakly irreducible tensors for any $\epsilon>0$. Now, we claim that $\mathcal{P}- \epsilon\mathcal{S}$ and $\mathcal {Q}- \epsilon\mathcal{S}$ are both strong *M*-tensors when $\epsilon >0$ is sufficiently small. Since $\mathcal{P}$ and $\mathcal{Q}$ are strong *M*-tensors, there exist positive diagonal matrices *C*, *D* such that

$$\mathcal{A}=\mathcal{P}\cdot C^{-(m-1)}\overbrace{C\cdots C}^{m-1},\qquad \mathcal{B}=\mathcal{Q}\cdot D^{-(m-1)}\overbrace{D \cdots D}^{m-1} $$

with

$$a_{i_{1}\dots i_{m}}=p_{i_{1}\dots i_{m}}c^{-(m-1)}_{i_{1}}c_{i_{2}} \cdots c_{i_{m}},\qquad b_{i_{1}\dots i_{m}}=q_{i_{1}\dots i_{m}}d^{-(m-1)}_{i_{1}}d_{i_{2}} \cdots d_{i_{m}}. $$

In particular,

$$a_{i\ldots i}=p_{i\ldots i},\qquad b_{i\ldots i}=q_{i\ldots i}. $$

By Lemma 2, one has

$$\begin{aligned}& |p_{i\ldots i}|=|a_{i\ldots i}|> \sum_{\delta _{{i}{i_{2}}\ldots{i_{m}}}=0}|p_{ii_{2}\dots i_{m}}|c^{-(m-1)}_{i}c_{i_{2}} \cdots c_{i_{m}}, \\& |q_{i\ldots i}|=|b_{i\ldots i}|> \sum_{\delta_{{i}{i_{2}}\ldots {i_{m}}}=0}|q_{ii_{2}\dots i_{m}}|d^{-(m-1)}_{i}d_{i_{2}} \cdots d_{i_{m}}. \end{aligned}$$

Set

$$L=\max_{\substack{i,j\in N\\ i\neq j}} \biggl\{ \frac {c^{[m-1]}_{j}}{c^{[m-1]}_{i}}, \frac{d^{[m-1]}_{j}}{d^{[m-1]}_{i}} \biggr\} $$

and

$$\begin{aligned} \epsilon_{0} =&\min_{ \substack{i,j\in N\\ i\neq j}} \biggl\{ \frac {|p_{i\ldots i}|-\sum_{\delta_{{i}{i_{2}}\ldots{i_{m}}}=0}|p_{ii_{2}\dots i_{m}}|c^{-(m-1)}_{i}c_{i_{2}}\cdots c_{i_{m}}}{(n-1)L}, \\ &\frac{|q_{i\ldots i}|-\sum_{\delta_{{i}{i_{2}}\ldots{i_{m}}}=0}|q_{ii_{2}\dots i_{m}}|d^{-(m-1)}_{i}d_{i_{2}}\cdots d_{i_{m}}}{(n-1)L} \biggr\} . \end{aligned}$$

Then for any $0<\epsilon<\epsilon_{0}$, it holds that $\mathcal {P}-\epsilon\mathcal{S}$ and $\mathcal{Q}-\epsilon\mathcal{S}$ are strong *M*-tensors. Substituting $\mathcal{P}-\epsilon\mathcal{S}$ and $\mathcal {Q}-\epsilon\mathcal{S}$ for $\mathcal{P}$ and $\mathcal{Q}$ and letting $\epsilon\to0$, we obtain the desired results by the continuity of $\tau(\mathcal{P}-\epsilon\mathcal{S})$ and $\tau (\mathcal{Q}-\epsilon\mathcal{S})$. □

Next, we give a lemma about the ratio of the smallest and largest values of a Perron vector for an irreducible nonnegative tensor.

### Lemma 6

(Lemma 3.2 of [35])

*Let* $\mathcal{B}$ *be a nonnegative irreducible tensor of order* $m\geq3$ *and dimension* *n* *with a Perron vector* *y*. *Then we have*

$$\kappa(\mathcal{B})\leq\frac{y_{\min}}{y_{\max}}, $$

*where* $\kappa(\mathcal{B})=\max_{2\leq k,k'\leq m} \min_{\substack{1\leq i_{1},i_{1'} \leq n\\1\leq i_{k}=i_{k'} \leq n}}\frac{\sum^{n}_{{\underbrace{i_{2},\ldots,i_{m}}_{\textit{except }i_{k}}}}b_{i_{1} i_{2}\ldots i_{m}}}{ \sum^{n}_{{\underbrace{i_{2'},\ldots,i_{m'}}_{\textit{except } i_{k'}}}}b_{i_{1'} i_{2'}\ldots i_{m'}}}$.

Based on the above lemma, we propose the following theorem, which provides a sharp bound under the condition of irreducibility.

### Theorem 2

*Suppose that* $\mathcal{P}$ *and* $\mathcal{Q}$ *are two irreducible strong* *M*-*tensors of order* *m* *and dimension* *n*, *and* $\rho(J_{\mathcal{P}})$ *and* $\rho (J_{\mathcal{Q}})$ *are their spectral radii with eigenvalue vectors* *u* *and* *v*, *respectively*. *Then*,

$$\tau(\mathcal{P}\star\mathcal{Q})\geq\min_{1\leq i,j\leq n,i\neq j}\biggl[1- \rho(J_{\mathcal{P}})\rho(J_{\mathcal{Q}}) +\frac{\alpha\beta |p_{i j\ldots j}|}{ p_{i\ldots i}}r^{j}_{i}(J_{\mathcal{Q}})+ \frac{\alpha \beta|q_{i j\ldots j}|}{ q_{i\ldots i}} r^{j}_{i}(J_{\mathcal {P}}) \biggr]p_{i\ldots i}q_{i\ldots i}, $$

*where* $\alpha=\kappa(J_{P})^{\frac{m-1}{2}}\leq [\frac{u_{\min}}{u_{\max }}]^{(m-1)}$, $\beta=\kappa(J_{Q})^{\frac{m-1}{2}}\leq[\frac{v_{\min }}{v_{\max}}]^{(m-1)}$, $r^{j}_{i}(J_{\mathcal{P}})=\sum_{\substack{ \delta_{{i}{i_{2}}\ldots{i_{m}}}=0\\ \delta_{{j}{i_{2}}\ldots{i_{m}}}=0}} \frac{|p_{i{i_{2}}\ldots{i_{m}}}|}{p_{i\ldots i}}$ *and* $r^{j}_{i}(J_{\mathcal{Q}})=\sum_{\substack{ \delta_{{i}{i_{2}}\ldots{i_{m}}}=0\\ \delta_{{j}{i_{2}}\ldots{i_{m}}}=0}}\frac{|q_{i{i_{2}}\ldots{i_{m}}}|}{q_{i\ldots i}}$.

### Proof

It follows from Lemma 4 that $\mathcal{P}\star \mathcal{Q}$ is a strong *M*-tensor. Since $\mathcal{P}$ and $\mathcal{Q}$ are strongly irreducible *M*-tensors, $J_{\mathcal{P}}$ and $J_{\mathcal{Q}}$ are irreducible nonnegative tensors. By the assumption that $\rho(J_{\mathcal{P}})$ and $\rho(J_{\mathcal{Q}})$ are the spectral radii with eigenvalue vectors *u* and *v*, we deduce that *u* and *v* are positive vectors such that

$$\rho(J_{\mathcal{P}})u^{[m-1]}_{i}=J_{\mathcal{P}}u^{m-1}, \qquad \rho (J_{\mathcal{Q}})v^{[m-1]}_{i}=J_{\mathcal{Q}}v^{m-1}, $$

equivalently,

10 $$\begin{aligned}& \frac{\sum_{\substack{ \delta_{{i}{i_{2}}\ldots{i_{m}}}=0\\ \delta_{{j}{i_{2}}\ldots{i_{m}}}=0}}|p_{i{i_{2}}\ldots{i_{m}}}|u_{i_{2}}\cdots u_{i_{m}}}{p_{i\ldots i}u^{[m-1]}_{i}}=\rho(J_{\mathcal{P}})-\frac{|p_{i j \ldots j}|u^{[m-1]}_{j}}{p_{i\ldots i}u^{[m-1]}_{i}}, \end{aligned}$$

11 $$\begin{aligned}& \frac{\sum_{\substack{ \delta_{{i}{i_{2}}\ldots{i_{m}}}=0\\ \delta_{{j}{i_{2}}\ldots{i_{m}}}=0 }}|q_{i{i_{2}}\ldots{i_{m}}}|v_{i_{2}}\cdots v_{i_{m}}}{q_{i\ldots i}v^{[m-1]}_{i}}=\rho(J_{\mathcal{Q}})-\frac{|q_{i j \ldots j}|v^{[m-1]}_{j}}{q_{i\ldots i}v^{[m-1]}_{i}}. \end{aligned}$$

Let $z=(z_{i})$, where $z_{i}= u_{i} v_{i}\in\mathbb{R}_{++}$ for $i\in N$. Setting $\mathcal{U} = \mathcal{P} \star\mathcal{Q}$, for $i \in N$, by (10) and (11), we have

12 $$\begin{aligned} \bigl(\mathcal{U}z^{m-1}\bigr)_{i} =&p_{i\ldots i}q_{i\ldots i}z_{i}^{[m-1]}-|p_{i j\ldots j}q_{i j\ldots j}|v_{j}^{[m-1]}u_{j}^{[m-1]} \\ &{} -\sum_{\substack{\delta_{{i}{i_{2}}\ldots{i_{m}}}=0\\ \delta_{{j}{i_{2}}\ldots{i_{m}}}=0}} |p_{i i_{2}\ldots i_{m}}| |q_{i i_{2}\ldots i_{m}}|z_{i_{2}}\cdots z_{i_{m}} \\ \geq& p_{i\ldots i}q_{i\ldots i}z_{i}^{[m-1]}-|p_{i j\ldots j}q_{i j\ldots j}|v_{j}^{[m-1]}u_{j}^{[m-1]} \\ &{}-\biggl(\sum_{\substack{ \delta_{{i}{i_{2}}\ldots{i_{m}}}=0\\ \delta_{{j}{i_{2}}\ldots{i_{m}}}=0}} |p_{i i_{2}\ldots i_{m}}|u_{i_{2}} \cdots u_{i_{m}}\biggr) \biggl(\sum_{\substack{ \delta_{{i}{i_{2}}\ldots{i_{m}}}=0\\ \delta_{{j}{i_{2}}\ldots{i_{m}}}=0}}|q_{i i_{2}\ldots i_{m}}|v_{i_{2}} \cdots z_{i_{m}}\biggr) \\ =&p_{i\ldots i}q_{i\ldots i}z_{i}^{[m-1]}\biggl[1- \frac{|p_{i j\ldots j}q_{i j\ldots j}|u_{j}^{[m-1]}v_{j}^{[m-1]}}{p_{i\ldots i}q_{i\ldots i}u_{i}^{[m-1]} v_{i}^{[m-1]}} \\ &{}-\biggl(\rho(J_{\mathcal{P}})-\frac{|p_{i j \ldots j}|u^{[m-1]}_{j}}{p_{i\ldots i}u^{[m-1]}_{i}}\biggr) \biggl( \rho(J_{\mathcal{Q}})-\frac {|q_{i j \ldots j}|v^{[m-1]}_{j}}{q_{i\ldots i}v^{[m-1]}_{i}}\biggr)\biggr] \\ =&p_{i\ldots i}q_{i\ldots i}z_{i}^{[m-1]}\biggl[1- \rho(J_{\mathcal{P}}) \rho (J_{\mathcal{Q}})+\frac{|p_{i j\ldots j}|u_{j}^{[m-1]}}{p_{i\ldots i}u_{i}^{[m-1]}} \biggl( \rho(J_{\mathcal{Q}})-\frac{|q_{i j \ldots j}|v^{[m-1]}_{j}}{q_{i\ldots i}v^{[m-1]}_{i}}\biggr) \\ &{}+\frac{|q_{i j\ldots j}|v_{j}^{[m-1]}}{q_{i\ldots i}v_{i}^{[m-1]}} \biggl(\rho(J_{\mathcal{P}})-\frac{|p_{i j \ldots j}|u^{[m-1]}_{j}}{p_{i\ldots i}u^{[m-1]}_{i}}\biggr) \biggr]. \end{aligned}$$

From (10) and Lemma 6, we deduce

13 $$ \rho(J_{\mathcal{P}})-\frac{|p_{i j \ldots j}|u^{[m-1]}_{j}}{p_{i\ldots i}u^{[m-1]}_{i}}\geq\sum _{\substack{ \delta_{{i}{i_{2}}\ldots{i_{m}}}=0\\ \delta_{{j}{i_{2}}\ldots{i_{m}}}=0}} \frac{|p_{i{i_{2}}\ldots{i_{m}}}|}{p_{i\ldots i}} \frac{u^{[m-1]}_{\min }}{u^{[m-1]}_{\max}}= \alpha r^{j}_{i}(J_{\mathcal{P}}). $$

Similarly,

14 $$ \rho(J_{\mathcal{Q}})-\frac{|q_{i j \ldots j}|v^{[m-1]}_{j}}{q_{i\ldots i}v^{[m-1]}_{i}}\geq\sum _{\substack{ \delta_{{i}{i_{2}}\ldots{i_{m}}}=0\\ \delta_{{j}{i_{2}}\ldots{i_{m}}}=0}} \frac{|q_{i i_{2}\ldots i_{m}}|}{q_{i\ldots i}} \frac{v^{[m-1]}_{\min }}{v^{[m-1]}_{\max}}=\beta r^{j}_{i}(J_{\mathcal{Q}}). $$

Combining (12) with (13) and (14), we have

15 $$ \bigl(\mathcal{U}z^{m-1}\bigr)_{i}\geq \biggl[ \biggl(1-\rho(J_{\mathcal{P}})\rho(J_{\mathcal {Q}}) +\frac{\alpha\beta|p_{i j\ldots j}|}{ p_{i\ldots i}} r^{j}_{i}(J_{\mathcal{Q}})+\frac{\alpha\beta|q_{i j\ldots j}|}{q_{i\ldots i}} r^{j}_{i}(J_{\mathcal{P}})\biggr) (p_{i\ldots i}q_{i\ldots i}) \biggr]z^{[m-1]}_{i}. $$

It follows from (15) and Lemma 3 that

$$\tau(\mathcal{P}\star\mathcal{Q})\geq\min_{1\leq i,j\leq n,i\neq j}\biggl[1- \rho(J_{\mathcal{P}})\rho(J_{\mathcal{Q}}) +\frac{\alpha\beta |p_{i j\ldots j}|}{ p_{i\ldots i}}r^{j}_{i}(J_{\mathcal{Q}})+ \frac{\alpha \beta|q_{i j\ldots j}|}{ q_{i\ldots i}} r^{j}_{i}(J_{\mathcal {P}}) \biggr]p_{i\ldots i}q_{i\ldots i}. $$

 □

### Remark 1

The bound in Theorem 2 is sharper than the result of Theorem 1, since $\frac{\alpha\beta|p_{i j\ldots j}|}{ p_{i\ldots i}}r^{j}_{i}(J_{\mathcal {Q}})+\frac{\alpha\beta|q_{i j\ldots j}|}{ q_{i\ldots i}} r^{j}_{i}(J_{\mathcal{P}})\geq0$.

The following example exhibits the efficiency of Theorems 1 and 2.

### Example 2

Let $\mathcal{P}=(p_{ijk})$, $\mathcal{Q}=(q_{ijk})$ be two tensors of order 3 and dimension 3 with elements defined as follows:

$$\mathcal{P}=\bigl[P(1,:,:),P(2,:,:),P(3,:,:)\bigr],\qquad \mathcal {Q}= \bigl[Q(1,:,:),Q(2,:,:),Q(3,:,:)\bigr], $$

where

$$\begin{array}{c} P(1,:,:)=\left(\begin{array}{ccc} 3 & 0 & -\frac{1}{3}\\ 0 & -1 & 0\\ -\frac{1}{3} & 0 & -\frac{1}{2} \end{array}\right),\;P(2,:,:)=\left(\begin{array}{ccc} 0 & -1 & 0\\ -1 & 3 & 0\\ 0 & 0 & -\frac{1}{2} \end{array}\right),\\ P(3,:,:)=\left(\begin{array}{ccc} -\frac{1}{3} & 0 & -\frac{1}{2}\\ 0 & 0 & -\frac{1}{2}\\ -\frac{1}{2} & -\frac{1}{2} & 5 \end{array}\right),\;Q(1,:,:)=\left(\begin{array}{ccc} 3 & -1 & 0\\ -1 & 0 & 0\\ 0 & 0 & -\frac{1}{3} \end{array}\right),\\ Q(2,:,:)=\left(\begin{array}{ccc} -1 & 0 & 0\\ 0 & 4 & -\frac{1}{2}\\ 0 & -\frac{1}{2} & -\frac{1}{3} \end{array}\right),\;Q(3,:,:)=\left(\begin{array}{ccc} 0 & 0 & -\frac{1}{3}\\ 0 & -\frac{1}{2} & -\frac{1}{3}\\ -\frac{1}{3} & -\frac{1}{3} & 2 \end{array}\right). \end{array}$$

It is clear that $\min_{1\leq i\leq n}(p_{i\ldots i}q_{i\ldots i})=9$. By computations, we get

$$\rho(J_{\mathcal{P}})=0.6842, \qquad \rho(J_{\mathcal{Q}})=0.7328,\qquad \alpha =\kappa(J_{P})=0.3,\qquad \beta=\kappa(J_{Q})=0.3. $$

From Theorem 1, we have

$$\tau(\mathcal{P}\star\mathcal{Q})\geq\bigl(1-\rho(J_{\mathcal{P}}) \rho (J_{\mathcal{Q}})\bigr)\min_{1\leq i\leq n}(p_{i\ldots i}q_{i\ldots i})=4.4876. $$

According to Theorem 2, we obtain

$$\begin{aligned} \tau(\mathcal{P}\star\mathcal{Q}) \geq&\min_{1\leq i,j\leq n,i\neq j}\biggl[1- \rho(J_{\mathcal{P}})\rho(J_{\mathcal{Q}}) +\frac{\alpha\beta |p_{i j\ldots j}|}{ p_{i\ldots i}}r^{j}_{i}(J_{\mathcal{Q}})+ \frac{\alpha \beta|q_{i j\ldots j}|}{ q_{i\ldots i}} r^{j}_{i}(J_{\mathcal {P}}) \biggr]p_{i\ldots i}q_{i\ldots i} \\ =&4.9074. \end{aligned}$$

By making use of the information of the absolute maximum in the off-diagonal elements, we are at the position to establish the following theorem.

### Theorem 3

*Suppose that* $\mathcal{P}$ *and* $\mathcal{Q}$ *are two strong* *M*-*tensors of order* *m* *and dimension* *n* *and assume that* $\rho(J_{\mathcal{P}})$ *and* $\rho(J_{\mathcal{Q}})$ *are the corresponding spectral radii*. *Then*

$$\tau(\mathcal{P}\star\mathcal{Q})\geq\min_{i\in N} \bigl\{ p_{i\ldots i}q_{i\ldots i}- \bigl(\alpha_{i} \beta_{i} p_{i\ldots i}q_{i\ldots i}\rho (J_{\mathcal{P}}) \rho(J_{\mathcal{Q}}) \bigr)^{\frac{1}{2}} \bigr\} , $$

*where* $\alpha_{i}=\max_{\delta_{{i}{i_{2}}\ldots{i_{m}}}=0}|p_{i i_{2}\ldots i_{m}}|$ *and* $\beta_{i}=\max_{\delta_{{i}{i_{2}}\ldots{i_{m}}}=0}|q_{i i_{2}\ldots i_{m}}|$.

### Proof

The proof is broken into two cases.

Case 1. $\mathcal{P}$ and $\mathcal{Q}$ are both weakly irreducible. It follows from Lemma 4 that $\mathcal{P}\star\mathcal{Q}$ is a strong *M*-tensor. Since $J_{\mathcal{P}}$ and $J_{\mathcal{Q}}$ are weakly irreducible nonnegative tensors, by Lemma 1, there exist two positive eigenvectors $u=(u^{2}_{i})>0$, $v=(v^{2}_{i})>0$ such that

16 $$\begin{aligned}& \frac{\sum_{ \delta_{{i}{i_{2}}\ldots{i_{m}}=0}}|p_{i{i_{2}}\ldots{i_{m}}}|u^{2}_{i_{2}}\cdots u^{2}_{i_{m}}}{p_{i\ldots i}u^{2[m-1]}_{i}}=\rho(J_{\mathcal{P}}), \end{aligned}$$

17 $$\begin{aligned}& \frac{\sum_{ \delta_{{i}{i_{2}}\ldots{i_{m}}}=0}|q_{i{i_{2}}\ldots{i_{m}}}|v^{2}_{i_{2}}\cdots v^{2}_{i_{m}}}{q_{i\ldots i}v^{2[m-1]}_{i}}=\rho(J_{\mathcal{Q}}). \end{aligned}$$

Without loss of generality, assume that $u_{i}, v_{i}\in\mathbb{R}_{++}$. Let $z=(z_{i})$ with $z_{i}= u_{i} v_{i}\in\mathbb{R}_{++}$ and $\mathcal{U} = \mathcal{P} \star\mathcal{Q}$. By Cauchy–Schwartz inequality, for $1\leq i\leq n$, we have

18 $$\begin{aligned} \bigl(\mathcal{U}z^{m-1}\bigr)_{i} =&p_{i\ldots i}q_{i\ldots i}z_{i}^{[m-1]}- \sum_{\delta_{{i}{i_{2}}\ldots{i_{m}}}=0} |p_{i i_{2}\ldots i_{m}}| |q_{i i_{2}\ldots i_{m}}|u_{i_{2}}v_{i_{2}} \cdots u_{i_{m}}v_{i_{m}} \\ \geq& p_{i\ldots i}q_{i\ldots i}z_{i}^{[m-1]}-\sum _{\delta _{{i}{i_{2}}\ldots{i_{m}}}=0} |p_{i i_{2}\ldots i_{m}}|u_{i_{2}} \cdots u_{i_{m}} \sum_{\delta_{{i}{i_{2}}\ldots{i_{m}}}=0} |q_{i i_{2}\ldots i_{m}}|v_{i_{2}} \cdots v_{i_{m}} \\ \geq& p_{i\ldots i}q_{i\ldots i}z_{i}^{[m-1]}-\biggl( \sum_{\delta _{{i}{i_{2}}\ldots{i_{m}}}=0}|p_{i i_{2}\ldots i_{m}}|^{2}u^{2}_{i_{2}} \cdots u^{2}_{i_{m}}\biggr)^{\frac{1}{2}} \\ &{}\times\biggl(\sum _{\delta_{{i}{i_{2}}\ldots{i_{m}}}=0}|q_{i i_{2}\ldots i_{m}}|^{2} v^{2}_{i_{2}} \cdots v^{2}_{i_{m}}\biggr)^{\frac{1}{2}}. \end{aligned}$$

It follows from the definitions of $\alpha_{i}$, $\beta_{i}$ and (18) that

19 $$\begin{aligned} \bigl(\mathcal{U}z^{m-1}\bigr)_{i} \geq& p_{i\ldots i}q_{i\ldots i}z_{i}^{[m-1]}- \bigl(\alpha_{i}{p_{i\ldots i}\rho(J_{\mathcal {P}})u^{2[m-1]}_{i}} \bigr)^{\frac{1}{2}}\bigl(\beta_{i}{q_{i\ldots i}} \rho(J_{\mathcal {Q}}){v^{2[m-1]}_{i}}\bigr)^{\frac{1}{2}} \\ =&\bigl[p_{i\ldots i}q_{i\ldots i}- \bigl(\alpha_{i} \beta_{i} p_{i\ldots i}q_{i\ldots i}\rho(J_{\mathcal{P}}) \rho(J_{\mathcal{Q}}) \bigr)^{\frac {1}{2}}\bigr]z_{i}^{[m-1]}. \end{aligned}$$

Furthermore, using Lemma 3 and (19), one has

$$\tau(\mathcal{P}\star\mathcal{Q})\geq\min_{i\in N} \bigl\{ p_{i\ldots i}q_{i\ldots i}- \bigl(\alpha_{i} \beta_{i} p_{i\ldots i}q_{i\ldots i}\rho (J_{\mathcal{P}}) \rho(J_{\mathcal{Q}}) \bigr)^{\frac{1}{2}} \bigr\} . $$

Case 2. Either $\mathcal{P}$ or $\mathcal{Q}$ is weakly reducible. Similar to the proof of Theorem 1, we obtain the desired result. □

In what follows, we give inclusion relations between Theorems 1 and 3.

### Corollary 1

*Let* $\mathcal{P}$ *and* $\mathcal{Q}$ *be strong* *M*-*tensors of order* *m* *and dimension* *n*.

*If* $p_{i\ldots i}q_{i\ldots i}\rho(J_{\mathcal{P}})\rho(J_{\mathcal {Q}})\leq\alpha_{i} \beta_{i}$ *for* $i\in N$, *then*

20 $$ \min_{i\in N}\bigl(1-\rho(J_{\mathcal{P}}) \rho(J_{\mathcal{Q}})\bigr)p_{i\ldots i}q_{i\ldots i}\geq\min _{i\in N} \bigl\{ p_{i\ldots i}q_{i\ldots i}- \bigl( \alpha_{i} \beta_{i} p_{i\ldots i}q_{i\ldots i} \rho(J_{\mathcal{P}})\rho (J_{\mathcal{Q}}) \bigr)^{\frac{1}{2}} \bigr\} ; $$

*if* $p_{i\ldots i}q_{i\ldots i}\rho(J_{\mathcal{P}})\rho(J_{\mathcal {Q}})\geq\alpha_{i} \beta_{i}$ *for* $i\in N$, *then*

21 $$ \min_{i\in N}\bigl(1-\rho(J_{\mathcal{P}}) \rho(J_{\mathcal{Q}})\bigr)p_{i\ldots i}q_{i\ldots i}\leq\min _{i\in N} \bigl\{ p_{i\ldots i}q_{i\ldots i}- \bigl( \alpha_{i} \beta_{i} p_{i\ldots i}q_{i\ldots i} \rho(J_{\mathcal{P}})\rho (J_{\mathcal{Q}}) \bigr)^{\frac{1}{2}} \bigr\} . $$

### Proof

Observe that

22 $$ \bigl(1-\rho(J_{\mathcal{P}}) \rho(J_{\mathcal{Q}}) \bigr)p_{i\ldots i}q_{i\ldots i}=p_{i\ldots i}q_{i\ldots i}-p_{i\ldots i}q_{i\ldots i} \rho(J_{\mathcal {P}}) \rho(J_{\mathcal{Q}})). $$

When $p_{i\ldots i}q_{i\ldots i}\rho(J_{\mathcal{P}})\rho(J_{\mathcal {Q}})\leq\alpha_{i} \beta_{i}$, from (22), we see

$$\begin{aligned} & \bigl(1-\rho(J_{\mathcal{P}}) \rho(J_{\mathcal{Q}})\bigr)p_{i\ldots i}q_{i\ldots i} \\ &\quad =p_{i\ldots i}q_{i\ldots i}-\bigl(p_{i\ldots i}q_{i\ldots i} \rho(J_{\mathcal {P}}) \rho(J_{\mathcal{Q}})\bigr)^{\frac{1}{2}} \bigl(p_{i\ldots i}q_{i\ldots i}\rho(J_{\mathcal{P}}) \rho(J_{\mathcal{Q}}) \bigr)^{\frac{1}{2}} \\ &\quad \geq p_{i\ldots i}q_{i\ldots i}-(\alpha_{i} \beta_{i})^{\frac {1}{2}}\bigl(p_{i\ldots i}q_{i\ldots i} \rho(J_{\mathcal{P}}) \rho(J_{\mathcal {Q}})\bigr)^{\frac{1}{2}} \\ &\quad = p_{i\ldots i}q_{i\ldots i}- \bigl(\alpha_{i} \beta_{i} p_{i\ldots i}q_{i\ldots i}\rho(J_{\mathcal{P}}) \rho(J_{\mathcal{Q}}) \bigr)^{\frac{1}{2}}, \end{aligned}$$

which implies

$$\min_{i\in N}\bigl\{ p_{i\ldots i}q_{i\ldots i}\bigl(1- \rho(J_{\mathcal{P}}) \rho (J_{\mathcal{Q}})\bigr)\bigr\} \geq \min _{i\in N} \bigl\{ p_{i\ldots i}q_{i\ldots i}- \bigl( \alpha_{i} \beta_{i} p_{i\ldots i}q_{i\ldots i} \rho(J_{\mathcal {P}})\rho(J_{\mathcal{Q}}) \bigr)^{\frac{1}{2}} \bigr\} . $$

So, (20) holds.

If $p_{i\ldots i}q_{i\ldots i}\rho(J_{\mathcal{P}})\rho(J_{\mathcal {Q}})\geq\alpha_{i} \beta_{i}$ for $i\in N$, similar to the proof of (20), we obtain (21). □

### Remark 2

If $p_{i\ldots i}q_{i\ldots i}\rho(J_{\mathcal{P}})\rho(J_{\mathcal {Q}})\leq\alpha_{i} \beta_{i}$ for all $1\leq i\leq n$, from (20), we verify that the bound of Theorem 1 is sharper than that of Theorem 3. When $p_{i\ldots i}q_{i\ldots i}\rho(J_{\mathcal{P}})\rho(J_{\mathcal {Q}})\geq\alpha_{i} \beta_{i}$ for $i\in N$, from (21), we deduce that the bound of Theorem 3 is tighter than that of Theorem 1.

The following examples give numerical comparisons between Theorems 1 and 3.

### Example 3

Let $\mathcal{P}=(p_{ijk})$, $\mathcal{Q}=(q_{ijk})$ be defined in Example 2.

It is clear that $\min_{1\leq i\leq n}(p_{i\ldots i}q_{i\ldots i})=9$. By computations, we get

$$\rho(J_{\mathcal{P}})=0.6842,\qquad \rho(J_{\mathcal{Q}})=0.7328, \qquad \alpha _{1}=\alpha_{2}=\beta_{1}= \beta_{2}=1,\qquad \alpha_{3}=\beta_{3}=1/2. $$

Obviously, $p_{i\ldots i}q_{i\ldots i}\rho(J_{\mathcal{P}})\rho (J_{\mathcal{Q}})\geq\alpha_{i} \beta_{i}$ for $i=1,2,3$. From Theorem 1, we have

$$\tau(\mathcal{P}\star\mathcal{Q})\geq\bigl(1-\rho(J_{\mathcal{P}}) \rho (J_{\mathcal{Q}})\bigr)\min_{1\leq i\leq n}(p_{i\ldots i}q_{i\ldots i})=4.4876. $$

From Theorem 3, we have

$$\tau(\mathcal{P}\star\mathcal{Q})\geq\min_{i\in N} \bigl\{ p_{i\ldots i}q_{i\ldots i}- \bigl(\alpha_{i} \beta_{i} p_{i\ldots i}q_{i\ldots i}\rho (J_{\mathcal{P}}) \rho(J_{\mathcal{Q}}) \bigr)^{\frac{1}{2}} \bigr\} =6.8758, $$

So, the bound of Theorem 3 is tighter than that of Theorem 1.

### Example 4

Let $\mathcal{P}=(p_{ijk})$, $\mathcal{Q}=(q_{ijk})$ be two tensors of order 3 and dimension 3 with elements defined as follows:

$$\mathcal{P}=\bigl[P(1,:,:),P(2,:,:),P(3,:,:)\bigr],\qquad \mathcal {Q}= \bigl[Q(1,:,:),Q(2,:,:),Q(3,:,:)\bigr], $$

where

$$\begin{array}{c} P(1,:,:)=\left(\begin{array}{ccc} 3 & 0 & 0\\ 0 & 0 & -\frac{11}{4}\\ 0 & 0 & 0 \end{array}\right),\;P(2,:,:)=\left(\begin{array}{ccc} 0 & -2 & 0\\ 0 & 4 & 0\\ 0 & 0 & -\frac{1}{4} \end{array}\right),\\ P(3,:,:)=\left(\begin{array}{ccc} -3 & 0 & 0\\ 0 & -\frac{1}{4} & 0\\ 0 & 0 & 5 \end{array}\right),\;Q(1,:,:)=\left(\begin{array}{ccc} 3 & 0 & 0\\ 0 & -\frac{1}{4} & 0\\ 0 & 0 & -2 \end{array}\right),\\ Q(2,:,:)=\left(\begin{array}{ccc} 0 & 0 & 0\\ 0 & 4 & 0\\ -2 & 0 & 0 \end{array}\right),\;Q(3,:,:)=\left(\begin{array}{ccc} -\frac{1}{4} & 0 & 0\\ 0 & -2 & 0\\ 0 & 0 & 3 \end{array}\right). \end{array}$$

By computations, we get

$$\begin{aligned}& \rho(J_{\mathcal{P}})=0.7036,\qquad \rho(J_{\mathcal{Q}})=0.6458, \qquad \alpha _{1}=\frac{11}{4}, \\& \beta_{1}=2,\qquad \alpha_{2}=\beta_{2}=2, \qquad \alpha_{3}=3, \qquad \beta_{3}=2. \end{aligned}$$

From Theorem 1, one has

$$\tau(\mathcal{P}\star\mathcal{Q})\geq\bigl(1-\rho(J_{\mathcal{P}}) \rho (J_{\mathcal{Q}})\bigr)\min_{1\leq i\leq n}(p_{i\ldots i}q_{i\ldots i})= \bigl(1-\rho(J_{\mathcal{P}}) \rho(J_{\mathcal{Q}})\bigr) p_{1\ldots 1}q_{1\ldots1} =4.9104. $$

According to Theorem 3, we obtain

$$\begin{aligned} \tau(\mathcal{P}\star\mathcal{Q})&\geq\min_{i\in N}\bigl\{ p_{i\ldots i}q_{i\ldots i}-\bigl(\alpha_{i} \beta_{i} p_{i\ldots i}q_{i\ldots i}\rho (J_{\mathcal{P}}) \rho(J_{\mathcal{Q}})\bigr)^{\frac{1}{2}}\bigr\} \\ &=p_{1\ldots1}q_{1\ldots1}-\bigl(\alpha_{1} \beta_{1} p_{1\ldots1}q_{1\ldots 1}\rho(J_{\mathcal{P}}) \rho(J_{\mathcal{Q}})\bigr)^{\frac{1}{2}} =4.2674. \end{aligned}$$

Thus, the bound of Theorem 1 is tighter than that of Theorem 3.

## Conclusions

In this paper, we generalized important inequalities on the minimum eigenvalue for the Fan product from matrices to tensors. Based on characterizations of *M*-tensors, we proposed lower bound estimates on the minimum eigenvalue for the Fan product of two *M*-tensors. Finally, we gave some sufficient conditions to establish when particular inclusion relations hold.
